# Comparison of the Most Demanding Periods in Three Categories of Female's Soccer: A Double View from the Player and the Team

**DOI:** 10.5114/jhk/207258

**Published:** 2025-11-19

**Authors:** Beñat Erkizia, David Casamichana, Fabio Yuzo Nakamura, Eider Barba, Julen Castellano

**Affiliations:** 1 Real Sociedad Institute, Real Sociedad de Fútbol S.A.D., Donostia-San Sebastián, Spain.; 2 Physical Education and Sports Department, University of the Basque Country (UPV/EHU), Vitoria-Gasteiz, Spain.; 3 Research Center in Sports Sciences, Health Sciences and Human Development (CIDESD), University of Maia (UMAI), Maia, Portugal.

**Keywords:** female, GPS, European football, match demands, movement analysis

## Abstract

This study is the first to quantify most demanding periods (MDPs) at both the individual (player) and the team level across three teams. It provides reference values for three external load variables across four time windows (of 1, 3, 5 and 10 min). The findings revealed that Professional (PRO) and Reserve (RES) teams covered more distance than the Academy (ACA) team in a 1-min window (w1), with the PRO team also covering greater distances at >18 km•h^−1^ in the w1. Additionally, PRO and RES teams experienced more accelerations in 1- and 3-min windows, with the PRO team showing higher values than the RES team in the 5-min window (w5). Players in PRO and RES teams ran longer distances and performed more accelerations than those in the ACA team, especially in 1-, 3-, and 5-min windows. No significant differences in MDPs were observed between the first and second halves for any team, but significant differences in distance covered were noted for PRO and ACA players in specific time windows. The main findings highlight superior locomotor and mechanical responses in PRO and RES players compared to ACA players, particularly in shorter time windows (w1, w3, and w5).

## Introduction

Advances in human tracking technologies have markedly increased the ability to perform movement analyses in running-based team sports (Wehbe et al., 2014). In recent years, there has been substantial development in computer-assisted tracking technology to examine players’ activity during training and matches (Buchheit et al., 2014). Global Positioning System-derived variables—such as total distance covered, the number of accelerations and decelerations, high-speed running (Martins et al., 2023), sprint distance (Reverte-Pagola et al., 2024), high metabolic load distance, player load (Guitart et al., 2022), and maximum top speed (Prudholme et al., 2023)— along with other relative metrics, are commonly used to quantify the external load.

Several studies in recent years have analysed most demanding periods (MDPs) in official matches (Rico-González et al., 2022). MDPs could be defined as the most intense periods that respond to the external load and are influenced by complex interactions between individual, tactical-technical, and contextual factors (Lino-Mesquita et al., 2025). In their study, Lino-Mesquita et al. (2025) used different time windows (1, 2, 3, 4, 5 and 10 min) and different external load variables (distance, high-speed running distance, sprint distance, high metabolic load distance, number of accelerations and player load) as criteria to locate moments of maximum activity, knowing that the intensity manifested by soccer players during MDP in official matches decreases in all external load variables studied as the duration of play lengthens (Rico-González et al., 2022), especially in external load variables related to high intensity (Riboli et al., 2024). These time windows have similar duration to the training tasks analyzed by other authors (Clemente et al., 2021; Ouertatani et al., 2022; Sanchez-Sánchez et al., 2018; Santos et al., 2024) and need to be taken into account when prescribing training (Savolainen et al., 2023). Unfortunately, in top-class women’s soccer, research findings about MDPs are still lacking (Riboli et al., 2024), and this study is the first to investigate MDPs in three categories of women's soccer.

MDPs emerge spontaneously during the match caused by the complex interaction of individual, collective and contextual factors (Lino-Mesquita et al., 2025). For example, the match location and the match outcome do not appear to influence the most demanding periods (MDPs) in elite female soccer players (González-García et al., 2023). However, as the duration of the time intervals increases, differences between the first and second halves also become more pronounced (Casamichana et al., 2019). The contextual variable that seems to have the greatest influence on the most demanding periods during the competition is the field position, suggesting that MDPs are position-specific (González-García et al., 2023). Positional differences were observed in peak high-speed running distance (HSRD) with central attacking midfielders, wide midfielders and forwards typically covering more HSRD than central defenders and central defensive midfielders (Datson et al., 2023). Other authors (Novak et al., 2021) added that the amount of activity completed in the period immediately preceding the MDP was also an important factor. In fact, they believed that a player entering the field at the end of a match and with low prior activity might achieve a higher peak MDP value than the same player who played the entire match and completed relatively high activity throughout.

Soccer academies are an important component of elite soccer organisations, and their primary objective is to develop youth players for promotion to professional teams (Williams et al., 2000). In recent years, there has been an increase in the number of soccer talent development programmes aimed at increasing players’ likelihood of progressing from academies to high-level soccer (Gledhill et al., 2017; Saward et al., 2020). A recent study compared MDP in different age categories among youth female soccer players (U14 vs. U16) (Harkness-Armstrong et al., 2021); while older players (U16) were found to cover greater distances both on average throughout the match and in MDP of between 1 and 9 min, there were no differences between the two groups in 10-min time windows. Currently, there is a lack of research comparing teams from three different categories within the same professional soccer club to assess whether MDPs become more demanding with age and category progression.

Traditionally, the concept of MDPs has been associated with peak activity levels of individual players. However, this study focused on analyzing the team's MDPs—specifically, the periods during which the average demand on all 10 field players is at its highest during a match. This approach that recognizes the team as a whole can result in different demanding phases from MDPs of individual players. Understanding these collective high-demand periods provides valuable insights into the physical demands placed on the team during critical phases of play. Therefore, the objective of this study was to compare the individual and team MDPs in three teams of different categories of the same club, using three external load variables (total distance covered, distance covered at >18 km•h^−1^ and the number of accelerations at >3 m•s^−2^) in four time windows (1, 3, 5 and 10 min). Three hypotheses were formulated: first, teams of a higher category would show higher MDP values; second, MDP values of the player unit would be higher than those of the team; and third, as the time window extended, the value of MDPs would be lower.

## Methods

### Participants

A total of 60 female soccer players participated in the study, as follows: a professional team (PRO; n = 20; age: 22.9 ± 2.9 years; body height: 166.7 ± 6.3 cm; body mass: 62.0 ± 5.4 kg; 30–15 intermittent fitness test (IFT): 19.0 ± 1.0 km•h^−1^), reserve team (RES; n = 20; age 19.4 ± 2.3; body height: 165.4 ± 5.2 cm; body mass: 58.9 ± 4.6 kg; 30–15 IFT: 18.8 ±1.0 km•h^−1^), academy team (ACA; n = 20; age: 16.3 ± 1.4 years; body height: 166.6 ± 4.2 cm; body mass: 59.0 ± 6.3 kg; 30–15 IFT: 18.2 ± 0.7 km•h^−1^). Thirty-six official matches (Table 1) were recorded (PRO = 15, RES = 12 and ACA = 9), giving a total of 804 player MDP records or events (PRO = 317, RES = 267 and ACA = 220) and 72 team MDP records (PRO = 30, RES = 24 and ACA = 18) for each of the external load variables in each of the time windows and the average for each half, as previously used (Vescovi et al., 2014). Goalkeepers were excluded from the analysis. Additionally, only players who played for over 45 min were included in the data analysis. Players who did not meet this criterion were withdrawn from the study. The data were gathered under a clause of the players’ employment conditions under which they underwent daily assessments. Nonetheless, the study conformed to the Declaration of Helsinki and players gave their informed consent before taking part in the study. For the utilization of data, the authorization of the club was required, then the identities of players were anonymized. This study was approved by the Ethics Committee of the University of the Basque Country (UPV/EHU), Vitoria-Gasteiz, Spain (approval code: M10-2024-124; approval date: 21 May 2024).

**Table 1:** The number of the records of the MDPs in the three external load variables (total distance covered, distance covered at >18 km•h^−1^ and the number of accelerations at >3 m•s^−2^) in the four windows (1-, 3-, 5- and 10-min) and of the two units (player and team) in the six periods of the official match.

			Period					
Variable	W	unit	P0–15	P16–30	P31–45+	P45–60	P60–75	P75–90+
TD0	w1	Player	179 (22.3)	98 (12.2)	69 (8.6)	271 (33.8)	123 (15.3)	63 (7.9)
		Team	20 (31.3)	7 (10.9)	7 (10.9)	10 (15.6)	11 (17.2)	9 (14.1)
	w3	Player	207 (25.8)	92 (11.5)	47 (5.9)	318 (39.6)	79 (9.8)	60 (7.5)
		Team	25 (39.1)	5 (7.8)	4 (6.3)	16 (25.0)	7 (10.9)	7 (10.9)
	w5	Player	224 (28.0)	82 (10.3)	40 (5.0)	324 (40.5)	84 (10.5)	46 (5.8)
		Team	23 (35.9)	7 (10.9)	4 (6.3)	15 (23.4)	6 (9.4)	9 (14.1)
	w10	Player	270 (34.4)	58 (7.4)	18 (2.3)	336 (42.9)	56 (7.1)	46 (5.9)
		Team	29 (45.3)	4 (6.3)	1 (1.6)	16 (25.0)	6 (9.4)	8 (12.5)
TD18	w1	Player	128 (15.9)	115 (14.3)	102 (12.7)	271 (33.8)	110 (13.7)	77 (9.6)
		Team	12 (18.8)	11 (17.2)	11 (17.2)	10 (15.6)	9 (14.1)	11 (17.2)
	w3	Player	140 (17.4)	130 (16.2)	75 (9.3)	275 (34.3)	116 (14.5)	67 (8.3)
		Team	17 (26.6)	11 (17.2)	6 (9.4)	12 (18.8)	9 (14.1)	9 (14.1)
	w5	Player	145 (18.1)	128 (16.0)	72 (9.0)	300 (37.5)	84 (10.5)	71 (8.9)
		Team	14 (21.9)	14 (21.9)	6 (9.4)	12 (18.8)	8 (12.5)	10 (15.6)
	w10	Player	182 (23.2)	121 (15.4)	42 (5.4)	306 (39.0)	80 (10.2)	53 (6.8)
		Team	18 (28.1)	12 (18.8)	4 (6.3)	15 (23.4)	7 (10.9)	8 (12.5)
AC3	w1	Player	181 (22.1)	117 (14.3)	57 (7.0)	296 (36.1)	109 (13.3)	60 (7.3)
		Team	17 (26.2)	10 (15.4)	8 (12.3)	8 (12.3)	11 (16.9)	11 (16.9)
	w3	Player	186 (22.7)	100 (12.2)	69 (8.4)	306 (37.3)	108 (13.2)	51 (6.2)
		Team	22 (34.4)	7 (10.9)	5 (7.8)	10 (15.6)	9 (14.1)	11 (17.2)
	w5	Player	199 (24.3)	99 (12.1)	57 (7.0)	302 (36.9)	114 (13.9)	47 (5.8)
		Team	19 (29.7)	11 (17.2)	4 (6.3)	9 (14.1)	9 (14.1)	12 (18.8)
	w10	Player	188 (23.3)	121 (15.0)	46 (5.7)	308 (38.2)	102 (12.7)	41 (5.1)
		Team	23 (35.9)	10 (15.6)	1 (1.6)	8 (12.5)	13 (20.3)	9 (14.1)

Note: TD is total distance, TD18 is distance covered >18 km•h^−1^, AC3 is the number of accelerations >3 m•s^−2^; W is time windows: w1, w3, w5 and w10 (of 1, 3, 5, and 10 min, respectively)

### Measures

Three external load variables were analysed: total distance covered (TD), distance covered at >18 km•h^−1^ (TD18) and the number of accelerations at >3 m•s^−2^ (AC3). These intensity thresholds were similar to those applied in previous studies on female soccer players (Andersson et al., 2010; Krustrup et al., 2005). The value of variables were normalized to the minute, in order to be able to compare the time windows of different duration. Variables TD and TD18 were expressed in meters per minute and AC3 in the number of actions per minute.

### Design and Procedures

To establish the external MDPs for each half-match observation, we used the rolling average method, which entailed computing averages for a designated window or interval of consecutive data points as the window moved progressively through the dataset. This approach is common for assessing MDPs and has been used in several previous studies (de Dios-Álvarez et al., 2024; Fereday et al., 2020; Rico-González et al., 2022). The data were analysed separately for the first and second halves of each game, with both values being used in the analysis. The MDPs were recorded for two different units, the player and the team. To calculate the players' values, we took their individual records. To calculate the team values, we acted similarly, considering the time window with the highest demand for the team as a whole. Team MDPs referred to the period during a match when the average external load—measured by variables such as total distance (TD), high-speed running distance (TD18), and accelerations at >3 m•s⁻^2^ (AC3)—of the 10 field players reached its peak. For example, to calculate the team MDP for TD, the total distance covered by all 10 field players during that period was summed and divided by 10 (i.e., the ten players on the field) to compare it to the player unit. The players’ average values in the half matches were calculated (w45), and four time windows of 1, 3, 5 and 10 min were established (w1, w3, w5, and w10, respectively). This time-window duration has been used extensively in the literature (Rico-González et al., 2022), since this is used as a reference for designing training games (Martin-Garcia et al., 2019).

The match was divided into two halves (first and second) and into six 15-min periods, named for their corresponding match minutes (P0–15, P16–30, P31–45+, P45–60, P60–75, and P75–90+). Periods P31–45+ and P75–90+ also included any stoppage time added. This allowed us to know in which period of the match the MDP of each variable occurred, considering the different time windows.

Table 1 shows the distribution of all the records included in the study, distinguishing the variable analysed, the two units considered (team and player), the time windows chosen, and the periods into which the match was divided.

The study was conducted during the 2023–2024 competitive season. The external match load was collected using GPS devices (WIMU SPRO V.980, Almería, Spain), worn by players from the start of the warm-up to the end of the match. All players were already familiar with the use of the devices, since it was part of their daily monitoring routine. GPS devices were fitted to the upper back (between the shoulder blades) by means of an adjustable neoprene harness. After each game, the data were extracted to a computer and analysed using SPRO software version 2.2.2. All official match records were exported to an Excel spreadsheet where a dataframe was configured. The external load variables of all the windows of the two units, player and team, were then relativized to the minute, allowing the different time windows to be compared. Official matches in which a player was sent off were not included in the analysis. Each team used the same 1-4-3-3 formation across all matches; although the physical demands on the different positional groups vary during match play (Romero-Moraleda et al., 2021; Sausaman et al., 2019), even among youth players (Ramos et al., 2017), we opted not to sub-divide the players by the field position, since the small cluster size for each group would have limited the statistical power.

### Statistical Analysis

The results are presented as the mean ± standard deviation (SD) for the monitored locomotor (TD and TD18) and mechanical (AC3) variables in the MDPs, for the two units (player and team), in all windows in the two halves of the official match and in three teams. Normality distributions were identified using both the Shapiro-Wilk test and the Kolmogorov-Smirnov test. ANOVA was used to determine the difference among teams and windows with the Bonferroni post-hoc correction test. The paired *t*-test was used to compare halves. The following classifications were used to measure the magnitude of Cohen’s *d* (Hopkins et al., 2009): trivial (< 0.2), small (0.2 > / <0.6), moderate (0.6> / < 1.2), large (1.2 > / < 2.0) and very large ( > 2.0). The effect of magnitude was established by power analysis, with the following threshold set for Cohen’s *d*: the minimum ES for the PRO team was 0.37, for the RES team 0.40 and for the ACA team 0.44 (G*power version 3.1.9.6). All tests were performed with 95% confidence intervals. JASP (version 0.18.3) software was used for statistical analysis (JASP Team, 2024. https://jasp-stats.org/).

## Results

### MDP Comparison for the Variable TD

**Figure 1:**
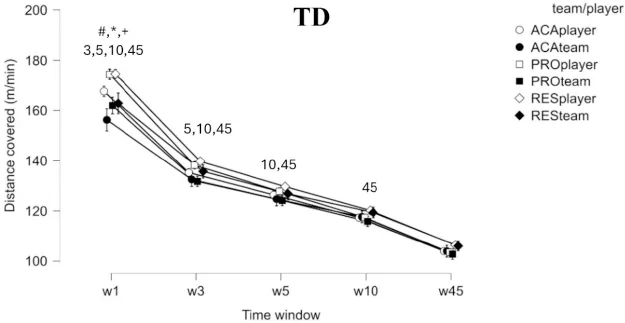
. Distance covered per min (TD) for the average of the player unit (PROplayer, RESplayer, and ACAplayer) and for the 10 outfield players from the PRO, RES, and ACA teams (professional team, reserve team and academy team, respectively) was calculated for each of the time windows, i.e., w1, w3, w5 and w10 (of 1, 3, 5, and 10 min, respectively), as well as the average for the entire half (w45). 3 is > w3, 5 is > w5, 10 is > w10 and 45 is > w45; # is >ACAteam, * is RESteam, and + is >PROteam (in each time window)

Among all teams, for both units, the player and the team, there were significant differences (*p* < 0.05) among time windows for the TD variable (Figure 1): w1 > w3 > w5 > w10 > w45, with a range in the ES from 0.71 to 5.71, except for: RESplayer in w10 = w45 and the windows w3 = w5 and w5 = w10 in the MDPs of the three teams (PROteam, RESteam, and ACAteam).

There were also significant differences (*p* < 0.05) among teams for the TD variable (Figure 1) in the w1: PROteam > ACAteam (ES = 0.86 [0.23/1.49]) and RESteam > ACAteam (ES = 0.76 [0.11/1.42]), and in three windows for players, in the w1: PROplayer > ACAplayer (ES = 0.55, 0.21/0.89) and RESplayer > ACAplayer (ES = 0.57, 0.21/0.92), in the w3: RESplayer > ACAplayer (ES = 0.36, 0.01/0.72), and in the w5: RESplayer > ACAplayer (ES = 1.11, 0.78/1.45). Finally, for the same time window, there were differences between the player and the team in the w1 for two of the three teams: PROplayer > PROteam (ES = 1.01, 0.2/1.81) and RESplayer > RESteam (ES = 0.95, 0.08/1.83), and only one for the w3: RESplayer > RESteam (ES = 1.04, 0.16/1.92).

### MDP Comparison for the TD18 Variable

**Figure 2:**
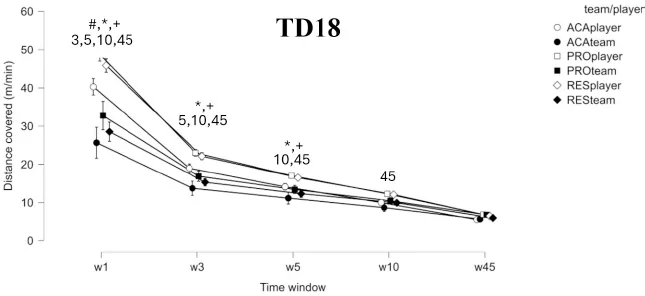
. The distance covered at speeds greater than 18 km•h^−1^ per min (TD18) was calculated for the average of the player unit (PROplayer, RESplayer, and ACAplayer) and for the 10 outfield players from the PRO, RES, and ACA teams (professional team, reserve team and academy team, respectively) in each of the time windows, i.e., w1, w3, w5 and w10 (of 1, 3, 5, and 10 min, respectively), as well as the average for the entire half (w45). 3 is > w3, 5 is > w5, 10 is > w10 and 45 is > w45; # is > ACAteam, * is RESteam and + is > PROteam (in each time window)

For the player unit, in all teams the time windows showed significant differences (*p* < 0.05) for the TD18 (Figure 2): w1 > w3 > w5 > w10 > w45, with a range in the ES from 0.35 to 5.01. However, for the team unit, there were no significant differences in the PROteam (w3 = w5 = w10 and w5 = w10 = w45) or in the RESteam and the ACAteam (w3 = w5 = w10 = w45).

For the TD18 variable (Figure 2), there were significant differences (*p* < 0.05) for the team unit in the w1: PROteam > RESteam, ES = 1.05 [−7.11 × 10^−3^/2.1] and PROteam > ACAteam, ES = 1.77 [0.65/2.89]. For the player unit, there were also significant differences among the three teams for the w1: PROplayer > RESplayer (ES = 0.35, 0.03/0.67), PROplayer > ACAplayer (ES = 1.01, 0.67/1.35), and RESplayer > ACAplayer (ES = 0.66, 0.31/1.02); for the w3: PROplayer > ACAplayer (ES = 0.48, 0.14/0.82) and RESplayer > ACAplayer (ES = 0.38, 0.02/0.73); and the w5: PROplayer > ACAplayer (ES = 0.35, 0.01/0.69). The following differences were found between units: in the w1, PROplayer > PROteam (ES = 1.92, 1.11/2.72), RESplayer > RESteam (ES = 2.07, 1.19/2.95), and ACAplayer > ACAteam (ES = 1.75, 0.81/2.7), and in w3, RESplayer > RESteam (ES = 1.18, 0.3/2.05).

### MDP Comparison for the AC3 Variable

**Figure 3:**
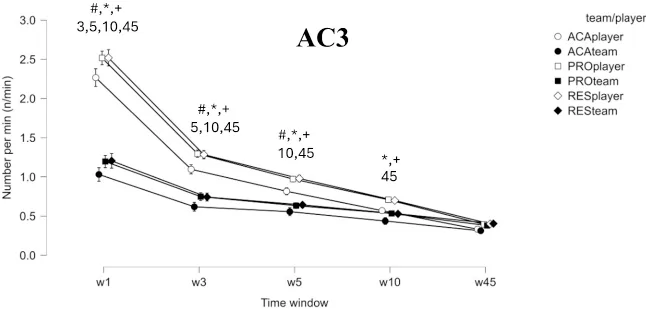
. The number of accelerations >3 m•s^−^^2^ per min (AC3) was calculated for both the average of the player unit (PROplayer, RESplayer, and ACAplayer) and for the 10 outfield players from the PRO, RES, and ACA teams (professional team, reserve team and academy team, respectively) in each of the time windows, i.e., w1, w3, w5 and w10 (of 1, 3, 5, and 10 min, respectively), as well as the average for the entire half (w45). 3 is > w3, 5 is > w5, 10 is > w10 and 45 is >w45; # is > ACAteam, * is RESteam and + is > PROteam (in each time window)

Finally, for the team unit, the greatest differences (*p* < 0.05) were found in the AC3 variable, especially in the first three windows (Figure 3): in the w1, PROteam > ACAteam (ES = 1.44, 0.32/2.57), RESteam > ACAteam (ES = 1.51, 0.32/2.71), in the w3, PROteam > ACAteam (ES = 1.28, 0.15/2.41, RESteam > ACAteam (ES = 1.15, −0.03/2.34), and in the w5, PROteam > ACAteam (ES = 0.89, −0.23/2.02). At the player level, there were also differences (*p* < 0.05) in three time windows (w1, w3 and w5) for PROplayer > ACAplayer (ES = 0.55, 0.21/0.9, ES = 0.44, 0.09/0.79 and ES = 0.34, − 0.02/0.69, respectively) and for RESplayer > ACAplayer (ES = 0.56, 0.20/0.92, ES = 0.41, 0.05/0.77, and ES = 0.36, 0.01/0.72, respectively).

When the two units, the player and the team, were compared, the latter outperformed the former in the following cases: for PROplayer > PROteam in the w1 (ES = 2.91, 2.14/3.68), the w3 (ES = 1.21, 0.43/1.98) and the w5 (ES = 0.75, −0.02/1.52), for RESplayer > RESteam in the w1 (ES = 2.9, 2.02/3.78) and the w3 (ES = 1.41, 0.54/2.29), and for ACAplayer > ACAteam in the w1 (ES = 2.72, 1.71/3.73) and in the w3 (ES = 1.05, 0.05/2.06).

#### MDP Comparison between Halves

Table 2 displays the mean and standard deviation of the three variables analyzed (TD, TD18, and AC3) for both the player and team units. These values were computed across four time windows (1, 3, 5, and 10 min), as well as for the first and second halves of the match. Statistical comparisons between the first and second halves revealed limited significant differences. Specifically, for TD, six significant differences were observed (w3 for ACA; w5 for PRO and ACA; w10 for PRO and ACA; and 45-min window for PRO). For TD18, only one significant difference was found (w1 for ACA in the player unit), while no significant differences were detected for AC3.

**Table 2:** Descriptive values (means and standard deviations) of the three external load variables analyzed in each half of the official match, in the four time windows and the three teams.

Variable			PRO		RES		ACA	
	W	unit	1^st^ half	2^nd^ half	1^st^ half	2^nd^ half	1^st^ half	2^nd^ half
TD	w1	Team	160.0 (± 7.7)	164.4 (± 8.1)	161.6 (± 7.3)	164.2 (± 10.7)	154.5 (± 9.9)	158.0 (± 8.0)
		Player	175.6 (± 18.4)	173.6 (± 19.1)	175.4 (± 14.7)	174.1 (± 15.3)	170.6 (± 13.8)	165.5 (± 16.5)
	w3	Team	134.0 (± 4.3)	128.8 (± 4.3)	137.7 (± 4.7)	133.5 (± 6.4)	132.4 (± 7.1)	132.5 (± 4.0)
		Player	140.5 (± 12.7)	136.3 (± 13.9)	142.5 (± 11.6)	137.8 (± 11.4)	138.7 (± 11.4)*	132.7 (± 11.1)
							ES = 0.7, 0.06/1.34	
	w5	Team	127.2 (± 3.0)	120.2 (± 4.0)	129.1 (± 4.4)	124.3 (± 5.4)	125.8 (± 6.5)	123.6 (± 4.0)
		Player	131.0 (± 11.4)*	125.0 (± 12.7)	132.4 (± 11.8)	127.7 (± 10.4)	129.9 (± 10.9)*	123.8 (± 10.8)
			ES = 0.71, 0.18/1.24				ES = 0.72, 0.08/1.36	
	w10	Team	118.8 (± 3.5)	112.1 (± 3.5)	121.2 (± 4.2)	117.4 (± 4.4)	118.7 (± 5.0)	116.6 (± 4.3)
		Player	120.9 (± 10.4)*	114.3 (± 11.7)	122.9 (± 12.7)	118.2 (± 10.0)	120.9 (± 10.4)*	114.8 (± 10.2)
			ES = 0.77, 0.24/1.31				ES = 0.72, 0.07/1.36	
	w45	Team	106.6 (± 3.2)	98.8 (± 4.7)	108.3 (± 3.8)	103.7 (± 4.4)	106.1 (± 4.8)	101.7 (± 2.9)
		Player	105.9 (± 11.2)*	101.4 (± 12.9)	108.3 (± 8.8)	105.0 (± 10.6)	106.0 (± 8.8)	102.5 (± 10.4)
			ES = 0.53, −0.03/1.05					
TD18	w1	Team	32.3 (± 10.5)	33.3 (± 7.3)	29.5 (± 7.3)	27.4 (± 2.5)	26.0 (± 7.6)	25.3 (± 9.1)
		Player	49.4 (± 15.8)	48.2 (± 15.7)	48.1 (± 13.9)	44.2 (± 14.7)	44.2 (± 15.9)*	37.6 (± 16.0)
							ES = 0.78, 0.14/1.42	
	w3	Team	17.0 (± 3.8)	16.8 (± 2.6)	15.5 (± 1.7)	15.1 (± 2.6)	14.2 (± 3.9)	13.3 (± 3.9)
		Player	23.7 (± 8.0)	22.4 (± 7.5)	23.2 (± 7.2)	21.4 (± 7.7)	20.7 (± 8.5)	17.8 (± 8.1)
	w5	Team	13.8 (± 2.9)	12.9 (± 2.1)	12.9 (± 1.8)	11.7 (± 2.2)	11.1 (± 2.7)	11.2 (± 3.6)
		Player	17.8 (± 6.2)	16.4 (± 5.9)	17.6 (± 5.9)	15.7 (± 5.7)	15.0 (± 6.2)	13.5 (± 6.4)
	w10	Team	10.5 (± 1.8)	10.4 (± 2.2)	10.2 (± 1.4)	9.6 (± 1.4)	8.7 (± 1.8)	8.7 (± 2.3)
		Player	12.7 (± 4.6)	11.9 (± 4.7)	12.7 (± 4.6)	11.6 (± 4.3)	10.8 (± 4.8)	9.5 (± 4.8)
	w45	Team	6.8 (± 1.1)	6.8 (± 1.3)	6.1 (± 1.0)	5.9 (± 1.4)	6.0 (± 1.4)	5.4 (± 1.0)
		Player	6.7 (± 2.8)	7.0 (± 3.9)	6.4 (± 2.7)	6.6 (± 3.4)	5.7 (± 3.0)	5.4 (± 3.2)
AC3	w1	Team	1.2 (± 0.2)	1.2 (± 0.2)	1.2 (± 0.2)	1.2 (± 0.2)	1.1 (± 0.1)	1.0 (± 0.2)
		Player	2.7 (± 0.8)	2.4 (± 0.8)	2.7 (± 0.8)	2.4 (± 0.9)	2.4 (± 0.8)	2.2 (± 0.8)
	w3	Team	0.8 (± 0.1)	0.8 (± 0.2)	0.8 (± 0.1)	0.7 (± 0.1)	0.7 (± 0.1)	0.6 (± 0.1)
		Player	1.4 (± 0.5)	1.2 (± 0.4)	1.4 (± 0.4)	1.2 (± 0.5)	1.2 (± 0.4)	1.0 (± 0.4)
	w5	Team	0.6 (± 0.1)	0.7 (± 0.1)	0.7 (± 0.1)	0.6 (± 0.1)	0.6 (± 0.1)	0.5 (± 0.1)
		Player	1.0 (± 0.3)	0.9 (± 0.3)	1.1 (± 0.3)	0.9 (± 0.4)	0.9 (± 0.3)	0.8 (± 0.3)
	w10	Team	0.5 (± 0.1)	0.5 (± 0.1)	0.6 (± 0.1)	0.5 (± 0.1)	0.5 (± 0.1)	0.4 (± 0.1)
		Player	0.7 (± 0.3)	0.7 (± 0.3)	0.7 (± 0.3)	0.7 (± 0.3)	0.6 (± 0.3)	0.5 (± 0.2)
	w45	Team	0.4 (± 0.1)	0.4 (± 0.1)	0.4 (± 0.0)	0.4 (± 0.1)	0.3 (± 0.1)	0.3 (± 0.0)
		Player	0.4 (± 0.1)	0.4 (± 0.2)	0.4 (± 0.2)	0.4 (± 0.2)	0.3 (± 0.2)	0.3 (± 0.2)

Note: Comparison of total distance (TD), distance at >18 km•h^−1^ (TD18), and the number of accelerations >3 m•s^−2^ (AC3) per min between the average of individual players (PROplayer, RESplayer, and ACAplayer) and the 10 outfield players from the PRO, RES, and ACA teams (professional team, reserve team and academy team, respectively) in each of the time windows (W): w1, w3, w5 and w10 (of 1, 3, 5, and 10 min, respectively), as well as the average for the entire half (w45). Effect size (ES) is reported as Cohen's d with 95% confidence interval. * indicates significant differences at p < 0.05 for 1^st^ half > 2^nd^ half

## Discussion

The main aim of the study was to compare the MDPs of locomotor (TD and TD18) and mechanical (AC3) performances among female soccer players belonging to three different competitive categories during official matches. Four time windows (1, 3, 5, and 10 min) for MDPs and both the first and second half averages were used to compare physical demands of the match. A novel aspect of this study is its dual perspective, taking both the player and the team as units of analysis. In the individual analysis, the focus for recording MDPs is for each player, as it has been proposed to date (Pillitteri et al., 2023). This corresponds to an isolated view of the players' punctual and maximum physical performance. However, in the team MDPs, all players have a high demand, which makes the physical performance of the team as a whole high. This collective view seems more interesting because it broadens the individual view to a collective one and, therefore, seems more relevant to have information with which to design training tasks aimed at the team as a whole. The main results of the study indicated that players in the first team (PRO) and the reserve team (RES) demonstrated superior locomotor and mechanical demand responses than those in the younger team (ACA), particularly in the shorter time windows (w1, w3, and w5).

The physical response of individual players was greater than the team average, especially in the one-minute period (w1), with these differences diminishing from the w3 onwards and disappearing altogether in the w5, the w10, and the w45. For all teams, players' activity levels varied across the time windows (w1 > w3 > w5 > w10 > w45) as in previous studies (Baptista el at., 2024; Fereday et al., 2020; González-García et al., 2023); however, this pattern was not observed in the overall team physical response, where the differences almost disappeared from the w3 onwards. There were no significant differences in the mechanical demands (AC3) of players between the first and second halves, which is contrary to other studies in which MDPs of acceleration distance and total distance declined from the first to the second half (Bradley, 2025; González-García et al., 2022). Lastly, there were only few differences in the total distance covered (TD) in 3- and 5-min windows; furthermore, these differences did not occur in all cases. Additional contextual factors (e.g., score, match importance, level of the opponent and weather conditions) should have been explored to further understand the complexity associated with the demands of the game (Bradley et al., 2025).

To the authors' knowledge, this is the first study to analyse MDPs in official matches from both the player and team perspectives. This novel approach is particularly interesting because it allows the average activity of the players and the team during training drills to be compared. Different studies have compared players’ activity during the MDPs of official matches with their activity during training games (Asian-Clemente et al., 2024; Martín-García et al., 2020; Rodríguez-Fernández et al., 2024). However, it is important to consider that individual MDPs in official matches occur at different moments of the game (Pillitteri et al., 2023). Therefore, understanding the team's activity levels at their peak periods is valuable for comparing them to the average demands of training matches. It is important to recognize that during these peak phases in official matches, some players will exhibit below-average values and others will register above-average figures, as occurs in training soccer games (Rabbani et al., 2024). Training tasks may not be designed to prepare athletes for the intense and multifaceted demands of MDPs (Novak et al., 2021). For this reason, coaches should be concerned with analyzing whether the intervention strategy they propose allows MDPs to emerge in training sessions. Large-sided games may be optimal for replicating full-distance MDPs in youth female soccer players, while small-sided formats (SSGs) seem to replicate AC3 MDPs, both in youth and senior players (Savolainen et al., 2023). However, small-sided game formats with specific sprinting rules, individualized positional drills, transition-sided games, or running-based exercises seem to be needed when the objective is to reproduce high-speed running values reached during MDPs (de Dios-Álvarez et al., 2024). The results of our study show that differences between the data for individual players and the team are more pronounced in short time windows, particularly in the w3 and even more so in the w1. This suggests that these short time windows likely present the least overlap in MDP timing between different players. Therefore, it is important to look for training strategies that replicate MDPs of the 1- and 3-min windows in the TD18 variable.

Several prior investigations (Riboli et al., 2024; Rico-González et al., 2022; Romero-Moraleda et al., 2021) have examined players’ activity across different teams within the same club. The objective was to discern potential disparities between teams, with the aim of facilitating players’ transfers between them. In our study, variations were observed in the short-duration MDPs in the total distance covered, with significantly lower values in the younger (ACA) team. Considering high-speed running (18 km•h^−1^), our observations revealed that as the age and the category of soccer players increased, the short-duration MDP became more demanding. As noted by Thoseby et al. (2023), the lower peak acceleration demands observed in youth competition might partly be explained by players’ age, the level of maturation, and possibly their reduced exposure to high-intensity competitive scenarios.

Previous research (Martín-García et al., 2019) has highlighted that the first halves, particularly the onset of the match (e.g., first 15 min), and, to a lesser degree, the commencement of the second half, represent the most physically demanding phases from a conditioning perspective (Saward et al., 2020). Nonetheless, a recent study highlights the need to include effective time in this analysis (Thoseby et al., 2023) when interpreting the match running performance of professional soccer players. By confining the depiction of conditional performance solely with very brief intervals such as those epitomized by the MDPs, players appear to be capable of replicating the demands across both halves (Trewin et al., 2018) even when confronted with successive matches during congested competition periods (Williams et al., 2000).

However, in the ACA team a decline was observed in some variables and within specific time frames. Notably, a critical objective in preparing youth players for the demands of professional soccer is to ensure they have the physical capabilities necessary for professional competitions (Sausaman et al., 2019). Those responsible for academies must therefore carefully consider implementing an appropriate long-term training regimen to effectively and gradually prepare players for the demands they will face.

Some of the main limitations of the study refer to the fact that contextual variables, such as the location (home and away), the result (win, draw or lose) and the opponents’ level, were not taken into account. Furthermore, due to the limited number of players in each team, the analysis did not consider the position occupied by the player on the pitch. Future studies should analyse the effective playing time of these MDPs, together with factors such as the number of possessions for each team, average duration of possession during these periods and the percentage of time spent in ball possession. Such analyses would undoubtedly help contextualize these periods more comprehensively.

## Conclusions

In conclusion, this study is the first to quantify the MDPs of three teams not only at the individual (player), but also at the team level. It offers reference values for three external load variables across four distinct time windows. The findings of this study highlight several key points. First, it was observed that the PRO and RES teams covered a greater distance than the ACA team in the w1. Also, the PRO team covered a greater distance at >18 km•h^−1^ than RES and ACA teams in the 1w. In addition, PRO and RES teams accelerated more times in the w1 and w3, while the PRO team accelerated more than the RES team in the w5. On the other hand, PRO and RES players ran longer distances than ACA players in the w1 and RES players also covered greater total distance than ACA players in the w3 and the w5. PRO players covered longer distance at >18 km•h^−1^ than RES and ACA players in 1- and 3-min windows, and more than ACA players in the w5. RES players also covered more distance than ACA players in the w1 and the w3, but not in the w5. PRO and RES players performed more accelerations than ACA players in 1-, 3- and 5-min windows.

Regarding the MDPs of the first and the second half, there were no significant differences in any of the three teams as a whole. There were significant differences in the distance travelled for PRO players in 5- and 10-min windows, and for ACA players in 3-, 5- and 10-min windows. Only ACA players significantly decreased their distance covered at >18 km•h^−1^ in the w1, and no player significantly decreased her performance in the number of accelerations at >3 m•s^−2^.

The main results of the study indicate that players in the first team (PRO) and the reserve team (RES) demonstrate superior locomotor and mechanical demands than those in the younger team (ACA), particularly in the shorter time windows (w1, w3, and w5).

## Practical Implications

The findings of this study offer several practical applications for coaches, strength and conditioning professionals, and academy managers in women's professional soccer. Firstly, the MDP data could be used to design training sessions that replicate the highest physical demands observed in official matches. Coaches can focus on high-intensity, short-duration drills, especially for players in the professional (PRO) and reserve (RES) teams, who exhibited higher activity levels during shorter time windows. This targeted training could help ensure that players are adequately prepared for peak demands during matches.

Secondly, understanding the differences in physical responses between individual players and the team as a whole, particularly in shorter time windows, would allow personalized training programmes to be developed. This is crucial for optimizing individual player performance and addressing specific conditioning needs. Additionally, the insights into physical demands across different team categories can guide the development of long-term training regimens in academies, ensuring young players (ACA) are effectively prepared for the physical challenges of professional soccer. These applications can enhance talent development, support strategic game planning, and ultimately improve overall team performance.
